# Endowed Beds with a History

**Published:** 1915-08-21

**Authors:** 


### ENDOWED BEDS WITH A HISTORY.

### I.—In the London Hospitals.

(FROM A CORRESPONDENT.)

In this and the following articles an account will be given of some of the more interesting of endowed beds and cots in London and the provinces, to many of which curious stories are attached. The writer takes this opportunity to thank the secretaries of the different hospitals for the trouble they have taken in looking up the details necessary for a fairly complete account.

### Germany and War Stories.

At the present time events give a topical interest to one of the beds in the New Hospital for Women, Euston Road, which is dedicated to the memory of the late German Emperor and his Empress. A tablet hangs in the board room, where I copied the wording, which is as follows: "A bed in this hospital has been endowed by subscription and dedicated to the use of patients suffering from cancer as a memorial to their late Imperial Majesties Frederick, German Emperor and King of Prussia, K.G., and Victoria, German Empress and Queen of Prussia, Princess Royal of Great Britain and Ireland, Duchess of Saxony." The present war, moreover, is responsible for a bed at the Miller General Hospital which has been endowed "In glorious memory of our brave soldiers and sailors fallen in the war, 1914."

"They feared not death."

"The path of duty was the way to glory."

When the war is over a further year will be added.

### Two Maiden Ladies and Guy's.

Guy's Hospital has two beds and two cots of special interest. One is endowed in memory of Miss Williams, an old lady over eighty years of age, who had received some kind attention through an old Guy's man of the name of Dr. Henry Pratt. Miss Williams desired to endow a bed in his memory. In this way she became interested in the hospital, and, after learning full particulars and making herself thoroughly acquainted with the whole management of the place, she died at the age of ninety-one, leaving to the hospital about £30,000. This provides a hint which other practitioners might take with profit to their "old" hospital!

The other bed is that endowed by a maiden lady, Miss Alicia Oliver, a native of Reading, who left England and went to live in France as a governess. She appeared to be in very poor circumstances, but some years before she died she consulted the British Consul in Dieppe, and, acting on his advice, she made a will and left all her income to be invested, providing an annuity for her sister, who predeceased her. The bequests in her will amounted to about £2,000, one of which was to endow a bed in the Reading Hospital and the other in Guy's Hospital. The British Consul wrote to the secretary explaining the difficulty he was in with regard to the realisation of the estate. So the secretary went over and assisted the Consul in the realisation of all the assets and brought back to Guy's Hospital the various securities, which were realised, and a bed was endowed in the name of the benefactress.

### Some Literary Memories.

Of the two cots in Guy's Hospital one is known as the Portsmouth "Tiny Tim" cot, which was endowed in memory of Charles Dickens by public subscription through the active energies of an old student by the name of Dr. Charles Hutt. The "Mary Lewin Henderson" cot was endowed by this lady, who conceived the idea of endowing a cot for the purpose of enabling the hospital to take in and care for little patients who needed hospital treatment, but she had no money for the purpose. She spent her spare time making fancy work, and induced as many of her friends as possible to become interested and purchase her needlework. Each year she handed over to the hospital a sum of money to be invested towards the required £500. After a labour of love for fifteen years she completed her collection and achieved her ambition by making the total contribution up to £500, and a cot was endowed in perpetuity. This good idea might commend itself to many people who cannot afford to endow a cot straight off.

St. Thomas's Hospital possesses a bed endowed anonymously in memory of Tom Hughes, the inscription being as follows: "March 1899. Anon. Tom Hughes bed. In memory of Tom Hughes, Q.C., author of 'Tom Brown's School Days.' Born 1823, died 1896."

Two "Melba" beds were endowed in 1908 at the London Hospital to commemorate the completion of twenty consecutive years as prima donna, when Madame Melba gave a matinée at Covent Garden, the proceeds of which—£2,000—she gave to the hospital.

The Royal Eye Hospital, Southwark, has a "Heroine" bed, named after a lady who lost the sight of one eye and by the proceeds of her knitting, etc., has contributed from time to time. She was called the heroine from the brave and courageous way in which she took her loss and for her efforts in making every possible use in her power to collect funds for the hospital.

### The Flower of Japan.

The late Duchess of Teck was much interested in the Chelsea Hospital for Women, and at her death it was decided to endow a bed in her memory by public subscription, to be known as the "Princess Mary" bed. When the endowment was complete our present Queen was pleased to accept all the privileges connected therewith as regards nominating patients, etc. At the *Dreadnought* Hospital is the "Chrysanthemum" bed, named in accordance with the wishes of the Japanese Ambassador when a donation of £500 was given to this hospital by the Emperor of Japan. The tablet for this bed was specially designed and executed in Japan, and is a very fine piece of work.

### An Out-patient Cot.

A cot in the Hospital for Epilepsy and Paralysis, Maida Vale, though only in process of endowment, I feel deserves a place here as an example of the love and self-denial which the poor so often show. I cannot do better than quote the secretary's words to me: "The courage and devotion of the parents in many cases is most praiseworthy. Fragile little mothers will trudge for miles with a child in a perambulator every day in all weathers so that the child shall have its treatment, and this in many cases goes on for years. One or two of these devoted women conceived the idea of a collecting-box scheme amongst themselves to raise sufficient to endow a cot, so that of the out-patients the one most deserving, either by reason of his medical needs, or the distance to be travelled to reach the hospital, or his own home circumstances, can always be accommodated. The patients are taking a keen interest in this collection, and one little boy aged six has already collected over £12 towards the fund, which now amounts to just over £50."

